# The impact of direct-maternal genetic correlations on international beef cattle evaluations for Limousin weaning weight

**DOI:** 10.1093/jas/skab222

**Published:** 2021-08-01

**Authors:** Renzo Bonifazi, Jérémie Vandenplas, Jan ten Napel, Roel F Veerkamp, Mario P L Calus

**Affiliations:** Animal Breeding and Genomics, Wageningen University and Research, P.O. Box 338, 6700 AH Wageningen, The Netherlands

**Keywords:** beef cattle, direct-maternal genetic correlation, international genetic evaluations, interbeef, international estimated breeding values, weaning weight

## Abstract

In beef cattle maternally influenced traits, estimates of direct-maternal genetic correlations (*r*_*dm*_) are usually reported to be negative. In international evaluations, *r*_*dm*_ can differ both within countries (*r*_*dm_WC*_) and between countries (*r*_*dm_BC*_). The *r*_*dm_BC*_ are difficult to estimate and are assumed to be zero in the current model for international beef cattle evaluations (Interbeef). Our objective was to investigate re-ranking of international estimated breeding values (IEBVs) in international beef cattle evaluations between models that either used estimated values for *r*_*dm*_ or assumed them to be 0. Age-adjusted weaning weights and pedigree data were available for Limousin beef cattle from ten European countries. International EBVs were obtained using a multi-trait animal model with countries modeled as different traits. We compared IEBVs from a model that uses estimated *r*_*dm_BC*_ (ranging between −0.14 and +0.14) and *r*_*dm_WC*_ (between −0.33 and +0.40) with IEBVs obtained either from the current model that assumes *r*_*dm_BC*_ to be 0, or from an alternative model that assumes both *r*_*dm_BC*_ and *r*_*dm_WC*_ to be 0. Direct and maternal IEBVs were compared across those three scenarios for different groups of animals. The ratio of population accuracies from the linear regression method was used to further investigate the impact of *r*_*dm*_ on international evaluations, for both the whole set of animals in the evaluation and the domestic ones. Ignoring *r*_*dm_BC*_, i.e., replacing estimated values with 0, resulted in no (rank correlations > 0.99) or limited (between 0.98 and 0.99) re-ranking for direct and maternal IEBVs, respectively. Both *r*_*dm_BC*_ and *r*_*dm_WC*_ had less impact on direct IEBVs than on maternal IEBVs. Re-ranking of maternal IEBVs decreased with increasing reliability. Ignoring *r*_*dm_BC*_ resulted in no re-ranking for sires with IEBVs that might be exchanged across countries and limited re-ranking for the top 100 sires. Using estimated *r*_*dm_BC*_ values instead of considering them to be 0 resulted in null to limited increases in population accuracy. Ignoring both *r*_*dm_BC*_ and *r*_*dm_WC*_ resulted in considerable re-ranking of animals’ IEBVs in all groups of animals evaluated. This study showed the limited impact of the current practice of ignoring *r*_*dm_BC*_ in international evaluations for Limousin weaning weight, most likely because the estimated *r*_*dm_BC*_ was close to 0. We expect that these conclusions can be extended to other traits that have reported *r*_*dm*_ values in the range of *r*_*dm_WC*_ values for weaning weight in Limousin.

## Introduction

In livestock, recorded traits can be influenced in their expression by the mother, i.e., they are influenced by maternal effects (Falconer and Mackay, 1996). Examples of such traits are growth and survival during the early stage of an animal’s life (Meyer, 2001; Knol et al., 2002; Hartmann et al., 2003; Eaglen and Bijma, 2009). Maternal effects reflect the mothers’ role in providing the environment to survive as well as nourishment for the offspring, starting from uterine development and continuing after birth until weaning (Meyer, 2001; Eaglen and Bijma, 2009), and have both a genetic and an environmental component (Falconer and Mackay, 1996). Therefore, in genetic evaluations of maternally influenced traits, the observed phenotypes are often dissected into a direct genetic effect, a maternal genetic effect, a maternal permanent environment effect, and into environmental effects common to siblings (Bijma, 2006; Mrode, 2014; Schaeffer, 2019). Maternal effects can contribute to phenotypic similarity in multiple offspring of the same dam, e.g., full-sibs and half-sibs, either arising from the same litter or different parities, and variability between families (Falconer and Mackay, 1996).

Models that account for direct and maternal genetic effects allow animal breeders to better estimate breeding values (EBVs) for these components, which are important for selection decisions and genetic progress of maternally influenced traits (Van Vleck et al., 1977; Gerstmayr, 1992; Mrode, 2014). Maternal effects are therefore usually included in the total merit indices for beef cattle (e.g., ICBF, 2020; Institut de l’Élevage, 2020) to reflect the maternal abilities of heifers and cows. Many studies have estimated the co-variance components of direct and maternal genetic effects in chickens, cattle, pigs, sheep, and rabbits (e.g., Hartmann et al., 2003; Koch, 1972; Knol et al., 2002; Abbasi et al., 2012; Krogmeier et al., 1994). The estimation of the magnitude of the genetic covariance and correlation between direct and maternal effects has been the object of study of animal breeders for a long time (Koch, 1972; Baker, 1980; Robinson, 1996a; Robinson, 1996b). In beef cattle, estimates of direct-maternal genetic correlations (*r*_*dm*_) are usually reported to be negative (Robinson, 1996a; Meyer, 1997). Estimates of *r*_*dm*_ could be subject to possible different sources of bias (Meyer, 1997; Clément et al., 2001; Bijma, 2006), and Meyer (1992) showed that large datasets are required for accurate estimation of genetic parameters of maternally affected traits. Later, Schaeffer (2019) suggested that three generations of female data are required to have a proper data structure for accurate estimations of *r*_*dm*_, and when this pedigree depth is not present, to set *r*_*dm*_ equal to 0 instead. Given the difficulties associated with the estimation of *r*_*dm*_, David et al. (2015) investigated the impact of ignoring *r*_*dm*_, on direct, maternal, and total EBVs (defined as the sum of direct and maternal EBVs) in sheep, pigs, and rabbits genetic evaluations. The authors showed that *r*_*dm*_ had a small influence on the total EBV, recommending, therefore, to set *r*_*dm*_ to 0 when their values are uncertain.

In the context of international evaluations, international EBVs (IEBVs) are computed across different environments (i.e., countries) for a series of traits (Durr and Philipsson, 2012; Crook et al., 2019). Some of the traits evaluated in beef international evaluations are influenced by maternal effects, e.g., birth weight, weaning weight, and calving ease (Phocas et al., 2005; Venot et al., 2006; Venot et al., 2007; Venot et al., 2008; Pabiou et al., 2014; Crook et al., 2019; Vesela et al., 2019). Due to the presence of genotype by environment interaction (i.e., genotype by country interaction), and differences in trait and model definition, genetic correlations (*r*_*g*_) for direct genetic effects, maternal genetic effects, and *r*_*dm*_ can differ between countries (De Mattos et al., 2000; Mark et al., 2005; Pabiou et al., 2014; Bonifazi et al., 2020b).

International genetic evaluations require estimates of across-country *r*_*g*_ (Phocas et al., 2005). However, the estimation process can be challenging (Mark et al., 2005; Venot et al., 2007), especially in beef cattle due to the low number of existing genetic connections between countries (Berry et al., 2016). These connections are established through animals having recorded offspring in more than one country, i.e., mainly international bulls (Jorjani et al., 2005; Bonifazi et al., 2020b). Moreover, this process is even more challenging for maternally influenced traits. Using large national datasets during this process allows to consider all existing genetic connections between countries at the expense of long, or even prohibitive, computational times (Bonifazi et al., 2020b). Therefore, data are usually reduced based on criteria that aim to maximize retained genetic connections across countries (Jorjani et al., 2005; Mark et al., 2005; Bonifazi et al., 2020b). Using a multi-country dataset, Bonifazi et al. (2020b) investigated the impact of data sub-setting on across-country *r*_*g*_ in beef cattle international evaluations led by Interbeef (2020) for Limousin weaning weight. While reducing data did not significantly affect across-country direct and maternal *r*_*g*_, within-country direct-maternal genetic correlations (*r*_*dm_WC*_) and between-country direct-maternal genetic correlations (*r*_*dm_BC*_) were more negative and were consistently affected by data reduction (−0.12 and −0.11 on average, respectively). When using all data, estimates of *r*_*dm_BC*_ were on average 0 and ranged from −0.14 to +0.14. However, they were most often not significantly different from zero, with standard errors being on average 0.14. France, however, had negative *r*_*dm_BC*_ (between −0.14 and −0.01) with all other countries except Switzerland which was the only country with a positive *r*_*dm_WC*_. Moreover, France represented 87% of the data and had the strongest connectedness with other countries. Four out of the 8 countries, including France, had an *r*_*dm_WC*_ that was significantly different from 0. These *r*_*dm_WC*_ being significantly different from 0 suggest that these estimates may be negative. Based on the estimates involving FRA, it seems that *r*_*dm_BC*_ do also have a tendency to be negative, albeit with relatively small deviations from zero.

In international evaluations, *r*_*dm_BC*_ are assumed to be zero since estimating them can be difficult (Venot et al., 2007; Vesela et al., 2019; Bonifazi et al., 2020b). However, little is known about the impact in international evaluations of assuming *r*_*dm_BC*_ and *r*_*dm_WC*_ to be zero. Therefore, our objective was, using previously estimated parameters, to investigate the impact on EBVs of using 0 instead of non-zero estimates for *r*_*dm*_ in the current international beef cattle evaluations model using Limousin weaning weight data. We studied the potential impact of this assumption on selection decisions by evaluating the re-ranking of different groups of animals, and the change in population accuracies and dispersion through the linear regression (LR) method (Legarra and Reverter, 2018).

## Materials and Methods

Animal Care and Use Committee approval was not requested for this study because commercial data were obtained from existing databases.

### Data and model

Age-adjusted weaning weight (AWW) phenotypes were available for 3,115,598 Limousin males and females, representing 8 Limousin populations and 10 European countries in the 2018 January Interbeef evaluation: Switzerland (CHE), Czech Republic (CZE), Germany (DEU), Denmark, Finland and Sweden (DFS, modeled as one population), Spain (ESP), France (FRA), Great Britain (GBR), and Ireland (IRL). Weaning weight was adjusted to an age of 210 days in CZE, ESP, and FRA and of 200 days in the remaining countries. Individual phenotypic records were distributed across 19,330 herds (Table 1). The majority (87%) of the observations available for AWW were from FRA, followed by GBR (4%), DFS, and DEU (about 3% each), whereas 1% or less of the total amount of records was from ESP, CHE, IRL, and CZE. Pedigree information were available for 3,431,742 animals, born between 1927 and 2017, with a maximum pedigree depth of 19 generations. A more detailed description of the data and pedigree editing criteria applied is provided in Bonifazi et al. (2020b). The number of direct and maternal genetic connections available in the pedigree was quantified in Bonifazi et al. (2020b). In total, 1,436 sires (also called “Common Bulls”—CB) and 3,828 maternal grand-sires (also called “Common MGS”—CMGS) had recorded offspring and grand-offspring in one or more countries, respectively. The number of CB ranged from 1,053 connecting 2 populations to 17 connecting 8 populations. The number of CMGS ranged from 3,040 connecting 2 populations to 19 connecting 8 populations. About 25% of the CMGS were also CB. Hereafter, for simplicity, we will refer to populations as countries, even though the DFS population is composed of more than one country.

**Table 1.** Summary of available age-adjusted weaning weights per country

COU^1^	AWW^2^	%^3^	Herds	YoB^4^	Pedigree^5^
CZE	10,500	0.3	121	1991–2017	30,843
DFS	90,456	2.9	9,190	1980–2017	117,623
ESP	33,152	1.1	188	1989–2011	63,526
GBR	127,840	4.1	745	1972–2017	172,229
IRL	20,609	0.7	1,304	1975–2017	56,694
FRA	2,714,368	87.1	6,677	1972–2017	2,942,297
DEU	88,628	2.8	881	1981–2017	121,228
CHE	30,045	1.0	224	1993–2017	55,104
Total	3,115,598	100	19,330	1972–2017	3,559,544

^1^Country: CZE, Czech Republic, DFS, Denmark, Finland and Sweden, ESP, Spain, GBR, Great Britain, IRL, Ireland, FRA, France, DEU, Germany, CHE, Switzerland.

^2^AWW, Age-adjusted weaning weight.

^3^ %, Percentage of AWW records per country.

^4^YoB, Year of birth of animals with records for AWW.

^5^Pedigree: number of animals retained in scenario NAT (national single-trait evaluations).

The AMACI model (Animal Model accounting for Across-Country Interaction) (Phocas et al., 2005) was used for the estimation of an animal’s breeding value, which accounts for country-specific fixed and random effects by fitting the national model of each country. The AMACI model is a multi-trait animal model with maternal effects, in which each country is modeled as a different trait:


$$\begin{array}{l} y_{i}=X_{i}b_{i}+C_{i}r_{i}+Z_{i}u_{i}+W_{i}m_{i}+P_{i}pe_{i}+\;e_{i} \end{array}$$

where *i* is the country; $y_{i}$ is the vector of observations for country *i*; $b_{i}$ and $r_{i}$ are the vectors of fixed and random environmental effects, respectively, for country *i* (as detailed below); $u_{i}$ and $m_{i}$ are the vectors of direct and maternal random additive genetic effects, respectively, for country *i* (i.e., corresponding to the vectors of IEBVs for each individual on each of the 8 country scales); $pe_{i}$ is the vector of random maternal permanent environmental effects (provided by the dam) for country *i*; and $e_{i}$ is the vector of random residual effects for country *i*. $X_{i}$ and $C_{i}$ are incidence matrices linking records to fixed and random environmental effects, respectively. $Z_{i}$, $W_{i}$, and $P_{i}$ are incidence matrices linking records to the animal, maternal genetic, and maternal permanent environmental effects, respectively. Fixed and random effects for each country are reported in Supplementary Table S1. In particular, random environmental effects were modeled for only three countries: CZE (herd-year-season), DEU (herd-year), and CHE (herd-year and sire-herd). The maternal permanent environmental effect was not fitted for the DEU population. It is assumed that


$$var\;\left[\begin{array}{c} u\;\\ m\; \end{array}\;\right]=\left[\begin{array}{cc} G_{d,d} & G_{m,d}\;\\ G_{d,m} & G_{m,m}\; \end{array}\right]⊗\;A$$

where **u** is the vector of random direct additive genetic effects for all countries; **m** is the vector of random maternal additive genetic effects for all countries; G is the across-country genetic (co)variance matrix of order 16 by 16 in which $G_{d,d}$ is the across-country direct additive genetic (co)variance matrix, $G_{m,m}$ is the across-country maternal additive genetic (co)variance matrix, and $G_{d,m}\;(G_{m,d})$ contains additive genetic covariances between direct and maternal effects within-country (diagonal elements) and additive genetic covariances between direct and maternal effects between-country (off-diagonal elements); **A** is the numerator relationship matrix; and ⊗ indicates the Kronecker product. Random environmental effects, random maternal permanent environmental effects, and residuals were fitted using block-diagonal variance matrices.

To closely represent current Interbeef evaluations, the genetic variance-covariance matrix with additive direct and maternal genetic effects (G) was built as


$$\begin{array}{l} G\;=\;S\;\Phi \;S \end{array}$$

where S is the diagonal matrix with national genetic standard deviations for direct and maternal genetic effects and Φ is the across-country estimated genetic correlation matrix (of order 16 × 16 with diagonal values of 1). The genetic correlation matrix Φ was previously estimated in Bonifazi et al. (2020b) (Scenario ALL) and was used for all scenarios implemented in this study. Both Φ and the obtained G (co)variance matrix are reported in Table 2. Both genetic and environmental variances were the same as those used in the national genetic evaluations of participating countries (Supplementary Table S2). Interbeef uses this procedure to compute the genetic variance–covariance matrix under the assumption that the national estimates of genetic variances are more accurate (e.g., when not all national data are submitted for international evaluations; Michenet, Interbull Centre, personal communication). The presence of genotype-by-country interactions due to differences in production systems and management conditions, as well as differences in trait and model definitions between countries, is accounted for in the AMACI model by modeling AWW of different countries as different correlated traits. These factors are reflected in estimated genetic correlations between countries lower than unity (Mark, 2004; Bonifazi et al., 2020b).

**Table 2.** Direct and maternal genetic correlations^1^ (below diagonal), genetic variances (diagonal), and covariances (above diagonal) within and across countries^2^

		DIRECT								MATERNAL							
		CZE	DFS	ESP	GBR	IRL	FRA	DEU	CHE	CZE	DFS	ESP	GBR	IRL	FRA	DEU	CHE
DIRECT	CZE	**310**	251.94	152.87	204.79	311.51	208.82	261.23	171.23	−28.53	−10.54	4.37	17.70	−7.52	−3.21	−6.45	14.89
	DFS	0.87	**269**	147.29	220.99	264.72	227.34	300.42	151.69	9.56	−24.69	12.65	7.85	14.99	−6.42	−25.95	12.76
	ESP	0.74	0.77	**136**	180.07	216.46	138.92	172.35	100.86	11.67	2.25	−21.18	−2.52	−10.30	−3.13	−5.53	5.77
	GBR	0.71	0.82	0.94	**268**	314.46	208.76	247.87	133.39	27.80	−2.47	−10.31	−11.63	−12.25	−7.68	−3.71	10.02
	IRL	0.83	0.76	0.87	0.91	**450**	252.19	257.72	169.97	−1.95	−5.61	−15.15	−4.68	−55.13	−14.71	24.25	4.72
	FRA	0.76	0.89	0.77	0.82	0.76	**242**	245.78	123.50	−22.60	−18.28	−6.22	−16.64	−25.56	−40.56	−27.93	−5.70
	DEU	0.76	0.94	0.76	0.77	0.62	0.81	**383**	157.07	20.83	−15.46	8.64	10.00	33.06	−1.72	−86.43	9.15
	CHE	0.85	0.81	0.76	0.71	0.70	0.70	0.70	**130**	1.87	−0.89	2.17	6.96	17.38	7.04	17.99	33.52
MATERNAL	CZE	−0.12	0.04	0.07	0.12	−0.01	−0.10	0.08	0.01	**197**	104.59	77.28	81.82	135.06	93.62	173.11	75.33
	DFS	−0.05	−0.14	0.02	−0.01	−0.02	−0.11	−0.07	−0.01	0.68	**120**	61.60	55.68	104.23	59.81	135.22	54.55
	ESP	0.03	0.09	−0.22	−0.08	−0.09	−0.05	0.05	0.02	0.67	0.68	**68**	42.66	93.59	46.17	100.20	40.46
	GBR	0.14	0.06	−0.03	−0.10	−0.03	−0.14	0.07	0.08	0.79	0.69	0.70	**55**	73.90	51.00	91.90	35.84
	IRL	−0.03	0.07	−0.06	−0.05	−0.19	−0.12	0.12	0.11	0.69	0.68	0.81	0.72	**194**	89.86	171.16	66.66
	FRA	−0.02	−0.05	−0.03	−0.06	−0.09	−0.33	−0.01	0.08	0.85	0.69	0.71	0.87	0.82	**62**	98.75	44.44
	DEU	−0.02	−0.09	−0.03	−0.01	0.06	−0.10	−0.24	0.09	0.68	0.68	0.67	0.69	0.68	0.69	**326**	87.49
	CHE	0.12	0.11	0.07	0.08	0.03	−0.05	0.06	0.40	0.73	0.68	0.67	0.66	0.65	0.77	0.66	**54**

^1^Genetic correlations originally reported in Bonifazi et al. (2020b).

^2^Country: CZE, Czech Republic, DFS, Denmark, Finland and Sweden, ESP, Spain, GBR, Great Britain, IRL, Ireland, FRA, France, DEU, Germany, CHE, Switzerland.

### Scenarios

Breeding values were estimated for the following scenarios, where *r*_*dm*_ within and between countries were either used or replaced by zero, while between-country direct and maternal *r*_*g*_ were used in all scenarios. This led to the following three scenarios, hereafter referred to as “international scenarios” (Table 3 summarizes the implemented (co)variance structure in each scenario):

**Table 3.** Fitted (●) variances and non-zero genetic correlations (*r*_*g*_) within and between countries per scenario^1,2^

	Scenario ^2^			
(co)variance structure	REF	CUR	NONE	NAT
Within-country direct and maternal variance	●	●	●	●
Between-country direct *r*_*g*_	●	●	●	
Between-country maternal *r*_*g*_	●	●	●	
Within-country direct-maternal *r*_*g*_	●	●		●
Between-country direct-maternal *r*_*g*_	●			

^1^ Not used *r*_*g*_ were replaced by 0.

^2^Scenario: NAT = national single-trait evaluations, NONE = both *r*_*dm_WC*_ and *r*_*dm_BC*_ set to 0, CUR = *r*_*dm_WC*_ used in the evaluation, and *r*_*dm_BC*_ set to 0, REF = both *r*_*dm_WC*_ and *r*_*dm_BC*_ used in the evaluation. With *r*_*dm_WC*_ = within-country direct-maternal genetic correlations, and *r*_*dm_BC*_ = between-country direct-maternal genetic correlations.

- Scenario REF: both *r*_*dm_WC*_ and *r*_*dm_BC*_ are used in the evaluation, i.e., the covariance structure across countries is as shown in Table 2. In this scenario, the information between direct and maternal breeding values is exchanged at the national level, through *r*_*dm_WC*_, and at an international level through *r*_*dm_BC*_. We considered this scenario as the reference scenario.
- Scenario CUR: *r*_*dm_WC*_ are used in the evaluation, but *r*_*dm_BC*_ are set to zero. This scenario represents the current-in-use methodology for Interbeef evaluations (Bonifazi et al., 2020b), where *r*_*dm*_ information is used at its minimum since information between direct and maternal breeding values is exchanged only at a national level through *r*_*dm_WC*_.
- Scenario NONE: both *r*_*dm_WC*_ and *r*_*dm_BC*_ are set to 0. In this scenario, there is no usage of *r*_*dm*_ and it was used as an extreme case to understand the effects of completely ignoring *r*_*dm*_.

Next to the international scenarios, breeding values were estimated with the following scenario aiming to represent pseudo-national single-trait evaluations:

- Scenario NAT: the AMACI model was run with all between-country *r*_*g*_ set to 0 (i.e., for *r*_*d*_, *r*_*m*_, and *r*_*dm_BC*_), while *r*_*dm_WC*_ were used. With this setting, the AMACI model is equivalent to run eight separate single-trait national evaluations. Once the model was run, for each country, EBVs were retained for all animals with phenotypes on their own, or any of their ancestors, or both. Hereafter, we will refer to those animals as the domestic set of animals. The number of retained domestic animals per country is reported in Table 1.

It should be noted that the resulting genetic correlation matrix in scenario CUR was non-positive definite, and therefore, it was bended using the unweighted bending approach of Jorjani et al. (2003) (using the R package “mbend” [Nilforooshan, 2020] with a threshold of 10^−3^). Initial analyses showed that the effects of bending on all the estimated *r*_*g*_ were minimal with an unweighted bending approach and allowed to keep the *r*_*dm_BC*_ close to 0. Moreover, bending the genetic correlation matrix allows to keep the same national genetic variances between international scenarios, which is often desired in international evaluations (Jorjani et al., 2003; Bonifazi et al., 2020b). Due to the bending process, some *r*_*dm_BC*_ showed small deviations from the fixed value of zero: ranging from −0.03 to 0.02. Changes due to bending in direct *r*_*g*_, maternal *r*_*g*_ and *r*_*dm_WC*_ ranged between −0.03 and 0.02, −0.03 and 0.01, and −0.04 and 0.09, respectively.

### Groups of animals evaluated

Changes in across-country *r*_*g*_ may result in re-ranking of animals, e.g., top bulls. We computed Spearman rank correlations of direct and maternal IEBVs for different groups of animals between the tested international scenarios that all used the full international pedigree:

1. All (3,431,742) animals included in the international evaluation.
2. Reliability (REL) class. Three groups of animals were formed based on their direct and maternal IEBV REL: REL ≤ 0.3, 0.3 < REL ≤ 0.6, and REL > 0.6. Approximated REL were computed using Tier and Meyer (2004) methodology under scenario REF.
3. Common Bulls (CB). In the analyzed dataset, 1,436 CB, i.e., sires with recorded offspring in more than one country, were present. Re-ranking of CB is an indicator of the *r*_*dm_BC*_ effect on the IEBV of animals having recorded offspring in two or more countries.
4. Sires with publishable IEBVs. In order to be published across countries, sire’s IEBV should fulfil several conditions (Michenet, Interbull Centre, personal communication). Sire’s direct IEBV should be associated with the following: 1) a REL ≥ 0.5 on at least one country scale and 2) ≥ 25 recorded progeny across all countries. Sire’s maternal IEBV should fulfil the following conditions: 1) have a publishable direct IEBV; 2) an associated REL ≥ 0.3 on at least one country scale; and 3) ≥ 15 daughters with recorded progeny and ≥ 25 recorded grand-progeny from daughters across all countries. Sires with IEBVs that fulfil the above requirements under the scenario REF were selected.
5. Young sires. Sires that fulfil the following conditions under the scenario REF were selected: 1) a publishable direct IEBV (following the same rules as described in group 4) and 2) a REL associated with a maternal IEBV < 0.3 on all country scales.

For each of the above groups of animals, we considered that no re-ranking was present between scenarios REF and CUR, and scenarios REF and NONE when the rank correlation was equal to or greater than 0.990. When the rank correlation was between 0.980 and 0.990, we considered that small re-ranking was present. Furthermore, for each scenario, we selected the top 100 sires on each country scale from those with a publishable IEBV (following the same rules as described in group 4) and calculated the number of commonly selected top 100 sires between scenarios.

### Increases in population accuracies and dispersion

To investigate the impact of modeling across-country *r*_*dm*_ on the population accuracies (acc), we used the LR method (Legarra and Reverter, 2018). Population accuracy is defined as the correlation between the true breeding values (TBVs) and the EBVs across individuals in a population (Legarra and Reverter, 2018). Following Legarra and Reverter (2018), the ratio of population accuracies of two evaluations, evaluation *p* with partial information, and evaluation *w* with all (i.e., whole) information is defined as $\rho_{p,w}=\;\frac{acc_{p}}{acc_{w}}$ and is computed as the Pearson correlation between EBVs from evaluations *p* and *w*,


$$\rho_{p,w}=\;\;\;\frac{{\left({\hat{u}}_{p}-{\bar{\hat{u}}}_{p}\right)}^{\prime }\;\left({\hat{u}}_{w}-{\bar{\hat{u}}}_{w}\right)}{\sqrt{{\left({\hat{u}}_{w}-{\bar{\hat{u}}}_{w}\right)}^{{}^{\prime }\;}\left({\hat{u}}_{w}-{\bar{\hat{u}}}_{w}\right){\left({\hat{u}}_{p}-{\bar{\hat{u}}}_{p}\right)}^{\prime }\left({\hat{u}}_{p}-{\bar{\hat{u}}}_{p}\right)}}$$

where ${\hat{u}}_{p}$ is the vector of animals’ EBVs from the partial evaluation and ${\hat{u}}_{w}$ is the vector of animals’ EBVs from the whole evaluation. Thus, $\rho_{p,w}$ is a direct estimator of the increase in population accuracy of EBVs from an evaluation with partial data to an evaluation with whole data (Legarra and Reverter, 2018).

In the context of international evaluations, national evaluations can be seen as evaluations with partial information, where recorded phenotypes are available only at the country level. International evaluations provide a new source of information through related animals being recorded in other countries and, therefore, represent evaluation *w*. Thus, the relative increases in population accuracy when moving from the partial (i.e., national; NAT) to the whole (i.e., international; INT) evaluations were calculated for each country and for international scenarios CUR and REF as the reciprocal of $\rho_{NAT_{d},INT_{d}}$ (Macedo et al., 2020), i.e., $\;1/\rho_{NAT_{d},INT_{d}}$, with $\rho_{NAT_{d},INT_{d}}\;=\;\frac{acc_{NAT_{d}}}{acc_{INT_{d}}}$, by using only domestic animals’ EBVs (denoted by *d*) of each country from scenario NAT, i.e., national animals. For example, when $\rho_{NAT_{d},INT_{d}}$ is 0.8, the additional information from the international evaluation increased the accuracy by 25% relative to the national evaluation ($1/\rho_{NAT_{d},INT_{d}}$ = 1.25).

The change in the *r*_*dm*_ structure implemented in the international scenarios can also be viewed as increasingly adding data to a “partial” international evaluation when moving from scenario NONE to CUR or REF, since non-zero values for the *r*_*dm*_ effectively change the amount of information contributing to the domestic direct and maternal animal’s IEBVs in a specific country. Thus, using all 3,431,742 IEBVs expressed on each country scale, we computed $1/\rho_{NONE,REF}$ and $1/\rho_{NONE,CUR}$. For instance, the latter represents the increase in population accuracy when fitting *r*_*dm_WC*_.

Using the LR method, we also evaluated any changes in dispersion of the EBVs between partial and whole evaluations. The estimator for dispersion (${\hat{b}}_{w,p}$) is defined as the slope of the regression of ${\hat{u}}_{w}$ on ${\hat{u}}_{p}$, computed as ${\hat{b}}_{w,p}=\;\frac{cov({\hat{u}}_{w},\;{\hat{u}}_{p})}{var({\hat{u}}_{p})}$ (Legarra and Reverter, 2018). The expectation of ${\hat{b}}_{w,p}$ is 1 if ${\hat{u}}_{w}$ and ${\hat{u}}_{p}$ have the same dispersion, while ${\hat{b}}_{w,p}$ > 1 indicates less dispersion in ${\hat{u}}_{p}$ compared to ${\hat{u}}_{w}$, and ${\hat{b}}_{w,p}$ < 1 indicates more dispersion in ${\hat{u}}_{p}$ compared to ${\hat{u}}_{w}$. We computed ${\hat{b}}_{w,p}$ between CUR or REF (whole evaluations) and NAT (partial evaluation) using only domestic animals’ EBV, and between REF (whole evaluation) and NONE or CUR (partial evaluations) using all IEBVs.

### Software and settings

In all scenarios, estimated breeding values and approximated reliabilities were obtained using MiX99 software (MiX99 Development Team, 2017). The convergence criterion of the preconditioned conjugate gradient (PCG) algorithm for the mixed-model equation solutions was defined as the square root of the relative difference between solutions of two consecutive PCG iterations and was set to 10^−7^. Convergence was also monitored for two other criteria, i.e., the relative difference between two consecutive solutions for the additive genetic animal effects and the relative residual of the mixed model equations.

## Results

First, we present the distribution of the EBV for the implemented scenarios, followed by the results on rank correlations for the different groups of animals evaluated and for the LR method.

### Distribution of EBV

**Figure 1.**
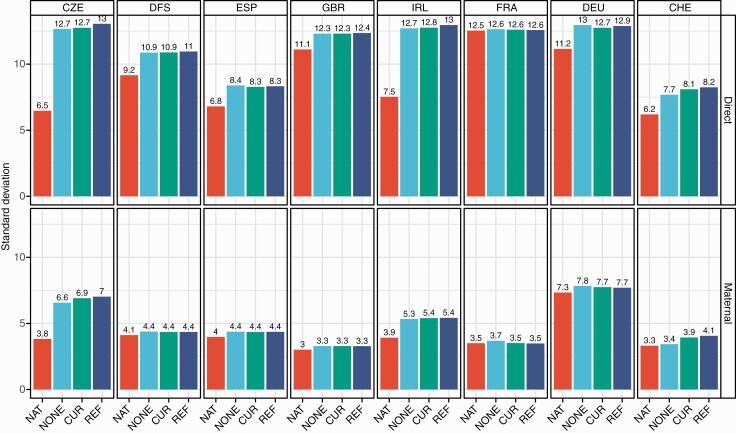
Direct and maternal estimated breeding value standard deviations of domestic animals^1^ per scenario^2^ on each country scale^3^. ^1^Domestic: animals retained in scenario NAT. ^2^Scenario: NAT = national single-trait evaluations, NONE = both *r*_*dm_WC*_ and *r*_*dm_BC*_ set to 0, CUR = *r*_*dm_WC*_ used in the evaluation, and *r*_*dm_BC*_ set to 0, REF = both *r*_*dm_WC*_ and *r*_*dm_BC*_ used in the evaluation. With *r*_*dm_WC*_ = within-country direct-maternal genetic correlations and *r*_*dm_BC*_ = between-country direct-maternal genetic correlations. ^3^Country: CZE = Czech Republic, DFS = Denmark, Finland and Sweden, ESP = Spain, GBR = Great Britain, IRL = Ireland, FRA = France, DEU = Germany, and CHE = Switzerland.

We computed the standard deviation (SD) across the EBV for the group of domestic animals for each country and scenario (Figure 1). The SD of domestic animals’ direct and maternal EBVs remained equal or increased when moving from national (NAT) to international evaluations (i.e., NONE, CUR, and REF) for all countries (Figure 1). The increase in EBV SD when comparing national with international evaluations was larger for smaller countries in terms of phenotypes (CZE, IRL, and CHE). CZE had the largest increase of EBV SD when moving from scenario NAT to REF, being 102% for direct EBV and 84% for maternal EBV. The SD of FRA domestic animals’ direct EBV remained almost the same under NAT and international scenarios, while slightly increased (5%) for maternal EBV under scenario NONE. In general, there were no large differences in terms of EBV SD between international scenarios.

### Impact on re-ranking of animals’ IEBV

Regarding the direct IEBV, no re-ranking was observed when considering all animals for all international evaluations in any country (rank correlations > 0.990). The only exceptions were for DEU and CHE for which small re-ranking was observed for the scenario NONE (i.e., rank correlations between REF and NONE ranged between 0.980 and 0.990; Table 4). Regarding the maternal IEBV, large re-ranking (i.e., rank correlations smaller than 0.980) could be observed across the different international scenarios. For example, the rank correlations between scenarios REF and NONE for maternal IEBVs of all animals were on average 0.965 across countries, with a minimum rank correlation of 0.917 for FRA (Table 4). The comparison between scenarios REF and CUR showed small re-ranking for maternal IEBVs, with an average rank correlation of 0.988 across countries, and a minimum rank correlation of 0.980 for DFS (Table 4).

**Table 4.** Spearman rank correlations of international estimated breeding values (IEBVs) between scenarios for different group of animals, and number of top 100 sires selected in common between scenarios

Scenario ^1^		COU ^2^	All ^3^		CB ^4^		Sires with publishable IEBVs^5^		Young sires ^6^		Top 100 sires	
			Direct	Maternal	Direct	Maternal	Direct	Maternal	Direct	Maternal	Direct	Maternal
REF	CUR	CZE	0.998	0.986	0.994	0.980	0.997	0.990	0.998	0.979	94	89
		DFS	0.998	0.980	0.993	0.988	0.997	0.990	0.999	0.968	92	84
		ESP	0.999	0.994	0.998	0.992	0.999	0.997	1.000	0.989	93	93
		GBR	0.999	0.984	0.997	0.985	0.999	0.992	0.999	0.973	93	88
		IRL	0.999	0.988	0.998	0.984	0.999	0.994	1.000	0.980	95	91
		FRA	1.000	0.992	0.999	0.996	1.000	0.997	1.000	0.985	99	94
		DEU	0.997	0.982	0.993	0.986	0.996	0.991	0.998	0.970	93	87
		CHE	0.997	0.995	0.993	0.994	0.995	0.997	0.997	0.992	91	91
REF	NONE	CZE	0.991	0.971	0.977	0.968	0.984	0.982	0.993	0.967	78	77
		DFS	0.991	0.976	0.978	0.978	0.986	0.987	0.994	0.962	82	80
		ESP	0.990	0.965	0.980	0.951	0.984	0.969	0.994	0.963	80	71
		GBR	0.994	0.973	0.987	0.973	0.989	0.987	0.996	0.956	85	84
		IRL	0.995	0.975	0.990	0.966	0.992	0.985	0.996	0.968	81	84
		FRA	0.998	0.917	0.998	0.952	0.999	0.971	0.999	0.831	97	78
		DEU	0.983	0.971	0.961	0.965	0.976	0.983	0.991	0.956	75	79
		CHE	0.982	0.972	0.966	0.967	0.971	0.977	0.986	0.975	73	75

^1^Scenario: NONE = both *r*_*dm_WC*_ and *r*_*dm_BC*_ set to 0, CUR = *r*_*dm_WC*_ used in the evaluation, and *r*_*dm_BC*_ set to 0, REF = both *r*_*dm_WC*_ and *r*_*dm_BC*_ used in the evaluation. With *r*_*dm_WC*_ = within-country direct-maternal genetic correlations, and *r*_*dm_BC*_ = between-country direct-maternal genetic correlations.

^2^COU, Country: CZE, Czech Republic, DFS, Denmark, Finland and Sweden, ESP, Spain, GBR, Great Britain, IRL, Ireland, FRA, France, DEU, Germany, CHE, Switzerland.

^3^All, All animals included in the international evaluation (i.e., 3,431,742).

^4^CB, Common bulls (1,436).

^5^Sires with publishable IEBVs: 32,208 direct IEBVs and 13,016 maternal IEBVs obtained under scenario REF.

^6^Young sires: 1,561 sires obtained under scenario REF.

For common bulls, re-ranking was more severe for maternal IEBVs than for direct IEBVs (Table 4). The comparison between scenarios REF and NONE showed re-ranking: rank correlations were on average 0.979 for direct IEBVs and 0.965 for maternal IEBVs, across countries. The comparison between scenarios REF and CUR showed no re-ranking for CB’s direct IEBV on any country scale (rank correlations > 0.990), and small re-ranking for maternal IEBV (rank correlations between 0.980 and 0.990) for CZE, DFS, GBR, IRL, and DEU (Table 4).

When grouped by their individual approximated reliabilities, the majority of the animals (> 78%) had a direct IEBV REL between 0.3 and 0.6, with the exception for CHE, where 64% of the animals had direct IEBV REL ≤ 0.3 (Supplementary Table S3). When grouped by maternal IEBV REL, almost all animals had a REL ≤ 0.6 and the majority of them had REL ≤ 0.3. FRA was the only country that had about 1% of the animals with maternal IEBV REL > 0.6 (Supplementary Table S3). Rank correlations between scenarios REF and NONE for direct IEBVs were 0.990, 0.990, and 0.985, for the three REL classes, respectively (Table 5). There was more variation in rank correlations between countries in the class of REL > 0.6 compared to the other two classes, with the smallest direct IEBV rank correlations for CHE (0.973) and DEU (0.975) (Table 5). There was no re-ranking between scenarios REF and CUR for direct IEBVs for any REL class on any country scale (all rank correlations > 0.997). Rank correlations between scenarios REF and NONE for maternal IEBVs increased with the REL class: average rank correlations across countries were 0.962, 0.968, and 0.980, respectively. Similarly, rank correlations between scenarios REF and CUR for maternal IEBVs increased with the REL class: average rank correlations across countries were 0.987, 0.991, and 0.995, respectively (Table 5).

**Table 5.** Spearman rank correlations of animals direct (Dir) and maternal (Mat) international estimated breeding values (IEBVs) between scenarios^1^ per class of reliability (REL)^3^, per country, and averaged across countries

Scenario ^1^		COU ^2^	Rank correlations					
			REL ≤ 0.3		0.3 < REL ≤ 0.6		0.6 < REL	
			Dir	Mat	Dir	Mat	Dir	Mat
REF	CUR	CZE	0.997	0.985	0.998	0.990	0.996	0.990
		DFS	0.996	0.980	0.998	0.987	0.997	0.998
		ESP	0.999	0.994	0.999	0.995	0.999	0.997
		GBR	0.999	0.983	0.999	0.990	0.999	0.993
		IRL	0.999	0.988	0.999	0.992	0.999	0.992
		FRA	0.999	0.989	1.000	0.995	1.000	0.998
		DEU	0.997	0.982	0.997	0.985	0.996	0.999
		CHE	0.997	0.995	0.997	0.997	0.995	0.996
		Average	0.998	0.987	0.998	0.991	0.997	0.995
REF	NONE	CZE	0.992	0.971	0.990	0.971	0.982	0.981
		DFS	0.993	0.976	0.991	0.978	0.986	0.993
		ESP	0.992	0.966	0.989	0.953	0.982	0.966
		GBR	0.995	0.972	0.994	0.979	0.990	0.988
		IRL	0.995	0.975	0.995	0.980	0.992	0.979
		FRA	0.993	0.892	0.998	0.947	0.996	0.974
		DEU	0.983	0.971	0.984	0.962	0.975	0.993
		CHE	0.981	0.973	0.981	0.971	0.973	0.967
		Average	0.990	0.962	0.990	0.968	0.985	0.980

^1^Scenario: NONE = both *r*_*dm_WC*_ and *r*_*dm_BC*_ set to 0, CUR = *r*_*dm_WC*_ used in the evaluation, and *r*_*dm_BC*_ set to 0, REF = both *r*_*dm_WC*_ and *r*_*dm_BC*_ used in the evaluation. With *r*_*dm_WC*_ = within-country direct-maternal genetic correlations, and *r*_*dm_BC*_ = between-country direct-maternal genetic correlations.

^2^COU, Country: CZE, Czech Republic, DFS, Denmark, Finland and Sweden, ESP, Spain, GBR, Great Britain, IRL, Ireland, FRA, France, DEU, Germany, CHE, Switzerland.

^3^REL computed under scenario REF.

The number of sires with publishable IEBVs was 32,208 and 13,016 for direct and maternal IEBVs, respectively. These sires were mainly recorded in FRA (89% and 90% of the total, for both direct and maternal IEBVs, respectively), followed by GBR, DFS, and DEU (each accounting for 2% to 4% of the total sires) and less than 1% in other countries (results not shown). The mean REL of sires with publishable IEBVs was 0.64 and 0.50 on average across countries for direct and maternal IEBVs, respectively (Supplementary Table S4). Re-ranking was present for sires with publishable IEBVs between scenarios REF and NONE for direct IEBVs on all country scales except for FRA and IRL: average rank correlation across countries of 0.985 and minimum rank correlation of 0.971 for CHE (Table 4). Similarly, re-ranking was present between scenarios REF and NONE for sires’ maternal IEBVs on all country scales: average rank correlation across countries of 0.980 and minimum rank correlation of 0.969 for ESP (Table 4). No re-ranking was observed, on any country scale, for sires with publishable IEBVs between scenarios REF and CUR, for both direct and maternal IEBVs (rank correlations > 0.995 and > 0.990, respectively; Table 4).

There were in total 1,561 young sires, the majority of which were recorded in FRA (78%), followed by GBR (9%), DFS (6%), DEU and CHE (2%), and other countries (1% or less) (results not shown). Young sires showed no re-ranking between scenarios REF and NONE for direct IEBVs, with the only rank correlations below 0.99 for CHE (0.986) (Table 4). Young sires showed re-ranking between scenarios REF and NONE for maternal IEBVs, with average rank correlations of 0.947 and minimum rank correlation of 0.831 for FRA. No re-ranking was observed for young sires between scenarios REF and CUR for direct IEBVs in any country (rank correlations > 0.997), while re-ranking was present for maternal IEBVs (average rank correlation of 0.979 and minimum rank correlation of 0.968 for DFS), with the exception of CHE (rank correlation of 0.992).

The number of commonly selected top 100 publishable sires between scenarios REF and NONE was 81 and 79 on average across countries for direct and maternal IEBVs, respectively (Table 4). The minimum number of commonly selected top 100 sires was 73 for CHE for direct IEBVs and 71 for ESP for maternal IEBVs. We further quantified the re-ranking between these scenarios using the absolute mean of the change in position of the list of top 100 sires selected under scenario REF when ranked based on their IEBVs under scenario NONE. Across countries, the top 100 sires moved on average by 16 and 23 positions, for direct and maternal IEBVs, respectively (Supplementary Table S5). The number of commonly selected top 100 publishable sires between scenarios REF and CUR was 94 and 90 on average across countries for direct and maternal IEBVs, respectively (Table 4). The minimum number of commonly selected top 100 sires was 91 for CHE for direct IEBV and 84 for DFS for maternal IEBV. Across countries, the top 100 sires, comparing scenario REF to CUR, moved on average by 2 and 5 positions, for direct and maternal IEBVs, respectively (Supplementary Table S5).

### Increases in population accuracies and dispersion

The increases in population accuracy when moving from scenario NONE to CUR or to REF, measured using $1/{\hat{\rho }}_{NONE,CUR}$ and $1/{\hat{\rho }}_{NONE,REF}$, respectively, are reported in Table 6. When comparing scenarios NONE and CUR, for direct IEBVs, $1/{\hat{\rho }}_{NONE,CUR}$ ranged from 0% (for CZE, DFS, GBR, IRL, and FRA) to 1% (for ESP, DEU, and CHE), while for maternal IEBVs, it was on average 2%, ranging from 1% (for DFS, IRL, and DEU) to 4% (for FRA) (Table 6). When comparing scenarios NONE and REF, $1/{\hat{\rho }}_{NONE,REF}$ was on average 1% for direct IEBVs across all countries, ranging from 0% (for FRA and IRL) to 2% (for CHE and DEU), while for maternal IEBVs, it was on average 3% across all counties, ranging from 2% (for GBR, IRL, and DFS) to 8% (for FRA) (Table 6). Comparison of $1/{\hat{\rho }}_{NONE,REF}$ with $1/{\hat{\rho }}_{NONE,CUR}$ shows similar gains of population accuracy when moving from scenario NONE to REF instead of moving to CUR, i.e., on average 0% for direct IEBVs and 1% for maternal IEBVs across countries. Only FRA benefits from using all correlations in the scenario REF in comparison to CUR (increase of 4%).

**Table 6.** Increase in population accuracy^1^ of moving from partial evaluation (*p)* to whole evaluation (*w*)^2^ for all animals included in the international evaluations (All) and domestic animals^3^

Effect	Scenario^4^		Country^5^							
All	Evaluation *p*	Evaluation *w*	CZE	DFS	ESP	GBR	IRL	FRA	DEU	CHE
Direct	NONE	CUR	0	0	1	0	0	0	1	1
	NONE	REF	1	1	1	1	0	0	2	2
Maternal	NONE	CUR	2	1	3	2	1	4	1	3
	NONE	REF	3	2	3	2	2	8	3	3
Domestic^**3**^	Evaluation *p*	Evaluation *w*								
Direct	NAT	CUR	59	6	19	4	35	0	9	21
	NAT	REF	63	7	20	4	38	0	10	23
Maternal	NAT	CUR	101	7	20	12	45	1	10	21
	NAT	REF	106	7	21	13	50	0	10	23

^1^Expressed as the relative % increase of evaluation *p*, i.e., $(1/{\hat{\rho }}_{p,w}-1)\;\cdot 100$.

^2^Partial: national evaluations (scenario NAT) where recorded phenotypes are available only at the country level.

^3^Whole: international evaluations (scenario CUR and REF) providing new information from other countries. Similarly, NONE is a partial evaluation relative to scenarios CUR and REF.

^3^Domestic: animals retained in the national evaluations for scenario NAT.

^4^Scenario: NAT = national single-trait evaluations, NONE = both *r*_*dm_WC*_ and *r*_*dm_BC*_ set to 0, CUR = *r*_*dm_WC*_ used in the evaluation, and *r*_*dm_BC*_ set to 0, REF = both *r*_*dm_WC*_ and *r*_*dm_BC*_ used in the evaluation. With *r*_*dm_WC*_ = within-country direct-maternal genetic correlations, and *r*_*dm_BC*_ = between-country direct-maternal genetic correlations.

^5^Country: CZE, Czech Republic, DFS, Denmark, Finland and Sweden, ESP, Spain, GBR, Great Britain, IRL, Ireland, FRA, France, DEU, Germany, CHE, Switzerland.

The increases in population accuracy for domestic animals obtained when moving from scenario NAT to CUR or REF are reported in Table 6. When comparing scenarios NAT and CUR, the increases in population accuracy for direct EBVs were on average 19% across countries, ranging from 0% of FRA to 59% of CZE. For maternal EBVs, the increases in population accuracy were on average 27% across countries, ranging from 1% of FRA to 101% of CZE. When comparing scenarios NAT and REF, the increases in population accuracy for direct EBVs were on average 21% across countries, ranging from 0% of FRA to 63% of CZE. For maternal EBVs, the increases in population accuracy were on average 29% across all countries, ranging from 0% of FRA to 106% of CZE. Comparison of $1/{\hat{\rho }}_{NAT_{d},REF_{d}}$ with $1/{\hat{\rho }}_{NAT_{d},CUR_{d}}$ shows the increase in population accuracy for domestic animals when moving from scenario NAT to REF instead of moving to CUR. For direct EBVs, differences of $1/{\hat{\rho }}_{NAT_{d},REF_{d}}$ with $1/{\hat{\rho }}_{NAT_{d},CUR_{d}}$ were on average 2%, ranging from 0% of FRA to 5% of CZE, while for maternal EBVs, differences were on average 2%, ranging from −1% for FRA to 5% of IRL.

Regression coefficients of IEBVs of whole on partial evaluations for all animals in the international evaluation are reported in Table 7. The regression coefficients ${\hat{b}}_{w,p}$ for direct IEBVs of REF-NONE were close to 1 in all countries, ranging from 0.99 of FRA to 1.06 of CHE. Direct IEBVs ${\hat{b}}_{w,p}$ of REF-CUR were also close to 1 in all countries, ranging from 1.00 of FRA to 1.04 of DFS and DEU. For maternal IEBVs, the ${\hat{b}}_{w,p}$ of REF-NONE were close to 1 in most of the countries, except for FRA and CHE for which ${\hat{b}}_{w,p}$ were 0.88 and 1.15, respectively. Maternal IEBVs ${\hat{b}}_{w,p}$ of REF-CUR were close to 1 in all countries, ranging from 0.96 of DFS to 1.06 of CHE.

**Table 7.** Direct and maternal regression coefficients of EBV of whole evaluation (*w*) on partial evaluation (*p*)^1^ (${\hat{b}}_{w,p}$) for all animals included in the international evaluation (All) and domestic^2^ animals

Effect	Scenario^3^		Country^4^							
All	Evaluation *p*	Evaluation *w*	CZE	DFS	ESP	GBR	IRL	FRA	DEU	CHE
Direct	NONE	REF	1.04	1.05	1.03	1.04	1.04	0.99	1.03	1.06
	CUR	REF	1.03	1.04	1.02	1.03	1.02	1.00	1.04	1.03
Maternal	NONE	REF	1.08	1.01	1.04	1.00	1.04	0.88	1.01	1.15
	CUR	REF	1.02	0.96	1.00	0.97	1.00	0.99	0.97	1.06
Domestic ^**2**^	Evaluation *p*	Evaluation *w*								
Direct	NAT	CUR	1.24	1.12	1.02	1.06	1.25	1.01	1.05	1.08
	NAT	REF	1.23	1.12	1.02	1.06	1.25	1.00	1.05	1.08
Maternal	NAT	CUR	0.90	0.99	0.91	0.97	0.95	0.99	0.96	0.98
	NAT	REF	0.89	0.99	0.91	0.97	0.92	0.99	0.95	0.99

^1^Partial: national evaluations (scenario NAT) where recorded phenotypes are available only at the country level. whole: international evaluations (scenario CUR and REF) providing new information from other countries. Similarly, NONE is a partial evaluation relative to scenarios CUR and REF.

^2^Domestic: animals retained in the national evaluations for scenario NAT.

^3^Scenario: NAT = national single-trait evaluations, NONE = both *r*_*dm_WC*_ and *r*_*dm_BC*_ set to 0, CUR = *r*_*dm_WC*_ used in the evaluation, and *r*_*dm_BC*_ set to 0, REF = both *r*_*dm_WC*_ and *r*_*dm_BC*_ used in the evaluation. With *r*_*dm_WC*_ = within-country direct-maternal genetic correlations, and *r*_*dm_BC*_ = between-country direct-maternal genetic correlations.

^4^Country: CZE, Czech Republic, DFS, Denmark, Finland and Sweden, ESP, Spain, GBR, Great Britain, IRL, Ireland, FRA, France, DEU, Germany, CHE, Switzerland.

Regression coefficients of EBVs of whole on partial evaluations for domestic animals are also reported in Table 7. The regression coefficients ${\hat{b}}_{w,p}$ for direct and maternal EBVs of NAT-CUR and NAT-REF were similar across countries. In NAT-CUR, direct EBVs ${\hat{b}}_{w,p}$ were close to 1 in almost all countries, ranging between 1.01 of FRA and 1.12 of DFS, except for CZE (1.24) and IRL (1.25). Similarly, direct EBVs ${\hat{b}}_{w,p}$ of NAT-REF ranged between 1.00 of FRA and 1.12 of DFS, except for CZE (1.23) and IRL (1.25). Maternal EBVs ${\hat{b}}_{w,p}$ of NAT-CUR were close to 1 in all countries, ranging between 0.90 of CZE and 0.99 of FRA. Similarly, maternal EBVs ${\hat{b}}_{w,p}$ of NAT-REF were close to 1 in all countries, ranging between 0.89 of CZE and 0.99 of FRA.

## Discussion

In this study, we investigated the impact of three different definitions of across countries *r*_*dm*_ on the ranking of international beef cattle IEBVs. We further explored the impact of *r*_*dm*_ on population accuracies and dispersion in international evaluations using the LR method. To the best of our knowledge, this is the first study that investigates the impact of the *r*_*dm*_ structure in the context of beef cattle international evaluations. Hereafter, we first discuss the impact of *r*_*dm_BC*_ and *r*_*dm_WC*_, followed by the possible implications of this study for beef cattle international evaluations.

### Impact of *r*_*dm_BC*_ on international evaluations

Ignoring *r*_*dm_BC*_ had a small impact on international evaluations. Ignoring *r*_*dm_BC*_ did not result in direct IEBV re-ranking for any country when considering all animals, while it did result in limited maternal IEBV re-ranking. This re-ranking was not associated with particular countries, but it was mainly related to animals with low maternal IEBV REL (REL ≤ 0.3). Ignoring *r*_*dm_BC*_ had also a small impact on all country scales for CB, with rank correlations between 0.980 and 0.990, while publishable sires showed no re-ranking on any country scale. In the latter group, Interbeef publication rules may have mitigated the impact of *r*_*dm_BC*_ by requiring sires to have records across two or three generations. As expected, young sires with low maternal IEBV REL (< 0.3) showed re-ranking for maternal IEBV on all country scales when *r*_*dm_BC*_ were ignored. Similarly, the impact of *r*_*dm_BC*_ on the re-ranking of the top 100 sires, assessed as the absolute mean change in ranking of the sires, was mostly limited to the maternal IEBV. Ignoring *r*_*dm_BC*_ had limited impact on domestic animals EBV SD: on average, the increase in EBV SD for scenario REF compared to CUR was 1% and 0% for direct and maternal EBVs, respectively, and mostly related to countries with smaller national populations (CZE, CHE, and IRL). These results are supported by the increases in population accuracy, when considering the whole set of IEBVs on each country scale, which hardly increased from using *r*_*dm_BC*_. Similar results were obtained for domestic animals: increases in population accuracy from modeling *r*_*dm_BC*_ were on average 2% for both direct and maternal EBVs, and at maximum 5% (associated with smaller countries such as CZE and IRL). These results were further confirmed by computing $1/{\hat{\rho }}_{CUR,REF}$ (results not shown) where no gain (0% for all countries) and maximum gains of 2% were obtained for direct and maternal EBVs, respectively. Similarly, regression coefficients ${\hat{b}}_{w,p}$ of REF-CUR were close to 1 in all countries for both direct and maternal IEBVs, indicating that IEBVs were not largely over- or under-dispersed, in comparison to REF, when ignoring *r*_*dm_BC*_. For domestic animals, corresponding regression coefficients ${\hat{b}}_{w,p}$ of REF-NAT and CUR-NAT were similar for both direct and maternal EBVs. Moreover, FRA had regression coefficients ${\hat{b}}_{w,p}$ close to 1 for both direct and maternal EBVs, while CZE and IRL showed the largest under-dispersion (${\hat{b}}_{w,p}$ > 1.23) of domestic animals’ national direct EBV compared to either CUR or REF. These results suggest that national EBVs in NAT may be under-dispersed compared to either CUR or REF international evaluations for smaller countries such as CZE and IRL. On the other hand, large countries like FRA showed no under- or over-dispersion (${\hat{b}}_{w,p}$ close to 1) of national EBVs relative to the international evaluations when data from other countries are considered. Thus, results from the LR method suggest that modeling *r*_*dm_BC*_ would not lead to large increases in population accuracy or dispersion in IEBV compared to ignoring them.

### Impact of *r*_*dm_WC*_ on international evaluations

Our results suggest that ignoring *r*_*dm_WC*_ affects international evaluations. Ignoring *r*_*dm_WC*_ resulted in re-ranking for both direct and maternal IEBVs. As expected, the largest impact on IEBVs re-ranking was observed for those countries with strong negative estimated *r*_*dm_WC*_, i.e., ESP and FRA. For example, FRA showed the lowest rank correlation for maternal IEBVs (0.917) when considering all animals, but grouping animals by their REL revealed that this re-ranking of FRA was mainly related to animals with REL ≤ 0.3. In general, when animals were grouped by REL, re-ranking within each group of REL was more severe in those countries with absolute values of *r*_*dm_WC*_ greater than 0.2 (i.e., FRA, ESP, DEU, and CHE). These results are in agreement with those of Phocas et al. (2004) which also observed an increased re-ranking for maternal IEBVs when ignoring *r*_*dm_WC*_ in international evaluations. Ignoring *r*_*dm_WC*_, in addition to *r*_*dm_BC*_, also gave more severe re-ranking for publishable sires and CB than ignoring only *r*_*dm_BC*_. Re-ranking in the latter group may be due to the majority of the CB originating from FRA (82.5%) (Bonifazi et al., 2020b). These FRA CB may have mainly recorded domestic offspring and, ignoring *r*_*dm_WC*_ for FRA, would lead to a different ranking for this group of animals on all country scales. Similarly, ignoring *r*_*dm_WC*_ led to high re-ranking of maternal IEBVs on all country scales for young sires, with the lowest rank correlation being for FRA. Re-ranking in this group may be related to the majority of young sires originating from FRA (78%). Ignoring *r*_*dm_WC*_ also affected the top 100 sires with publishable IEBVs, both for direct and maternal IEBVs. This was also confirmed by the results for the absolute mean change in position of top 100 sires. FRA contributes with the largest amount of data in the Limousin Interbeef evaluation (87% of all phenotypes) and has the strongest pedigree connections with other countries. Thus, ignoring *r*_*dm_WC*_ in FRA may have contributed to generating re-ranking for the categories of publishable sires, young sires, and CB not only on the FRA scale, but also on other countries scales since the majority of the animals in these categories originates from FRA. Ignoring *r*_*dm_WC*_ affected the increases of domestic animals EBV SD, for both small countries like CHE and CZE (increases in EBV SD of CUR compared to NONE higher than 5%), and large countries like FRA (maternal EBV SD 5% smaller in scenario NONE compared to CUR). The importance of using *r*_*dm_WC*_ in international evaluations was also reflected in the increases in population accuracy for the whole set of IEBVs. Similarly, regression coefficients ${\hat{b}}_{w,p}$ of REF-NONE show that ignoring *r*_*dm_WC*_, in addition to *r*_*dm_BC*_, led to over- or under-dispersion of maternal IEBVs compared to REF, especially in countries such as FRA and CHE where *r*_*dm_WC*_ were the strongest (absolute values > 0.3). On the other hand, when *r*_*dm_WC*_ were considered in the international evaluation, the difference in dispersion in comparison to REF reduced for almost all countries with values for regression coefficients ${\hat{b}}_{w,p}$ closer to 1. Van Vleck (1977) showed that when *r*_*dm*_ are negative, these correlations need to be considered for selection decisions. Thus, the results of our study support the suggestion of Phocas et. al. (2004) that when estimates of *r*_*dm_WC*_ are considerably different from 0, as was the case for most countries, they should be used in international evaluations and not ignored.

### Implications

Both *r*_*dm_BC*_ and *r*_*dm_WC*_ had less impact on the re-ranking of animals for direct IEBVs than for maternal IEBVs. For a young sire, the AWW record is available at an early stage of his life, while data recorded on his daughters and daughters’ progeny, necessary to make an accurate estimate of the maternal EBV, are available only later in his life (Willham, 1980; Gerstmayr, 1992). The maternal EBV of young sires is mainly based on its direct EBV through *r*_*dm_WC*_, while later in life it will be increasingly more based on daughters’ progeny records, and thus will be less affected by the *r*_*dm_WC*_. In international evaluations, the maternal IEBV of a domestic young sire with mostly recorded foreign offspring will heavily depend on its foreign direct IEBV through the *r*_*dm_BC*_. Therefore, re-ranking in maternal IEBV of young sires is expected as they become older and, considering both the large standard error of estimated *r*_*dm_BC*_ and the associated difficulties in estimating them (Bonifazi et al., 2020b), a ranking based on progeny’ record may be desired. These expected re-rankings were confirmed by the low rank correlations observed for maternal IEBVs for the group of young sires. Results of this study are in line with the findings of David et al. (2015) in other species who suggested that *r*_*dm*_ have a greater impact on maternal EBV since they are derived from offspring performance, as opposed to direct EBV. Our results showed that re-ranking of maternal IEBV decreased with increasing reliability, regardless of the *r*_*dm*_ structure used, suggesting that when more information is available at the animal level, the *r*_*dm*_ become less important for the estimation of maternal IEBVs. Another possible explanation for *r*_*dm*_ having a greater impact on maternal IEBV, as suggested by David et al. (2015), may be that national direct genetic variances were larger than maternal ones. On the other hand, our results confirmed that little or no re-ranking is expected when ignoring *r*_*dm_BC*_ for top 100 sires and sires with publishable IEBVs, respectively, because these sires have both recorded progeny and daughters’ offspring available. Thus, the results of this study provide support that fixing *r*_*dm_BC*_ to 0 has limited impact on IEBV for Limousin weaning weight.

Estimated values used for *r*_*dm_BC*_ tended to be negative, similarly to *r*_*dm_WC*_. The different *r*_*dm_BC*_ of FRA were consistently negative and, given the amount of phenotypes provided by FRA to the Limousin international evaluation, ignoring *r*_*dm_BC*_ potentially could affect international evaluations. However, our results show that the impact of ignoring *r*_*dm_BC*_ in international evaluations was limited, most likely because the absolute values of *r*_*dm_BC*_ were close to 0. Absolute values of *r*_*dm_BC*_ were smaller compared to absolute values of *r*_*dm_WC*_; this difference is expected, as relative to *r*_*dm_WC*_, the magnitude of *r*_*dm_BC*_ may be reduced due to genotype-by-country interactions. With stronger genotype-by-country interactions, we expect estimates of *r*_*dm_BC*_ to be closer to 0, reducing the impact of ignoring *r*_*dm_BC*_ on international evaluations. On the other hand, the presence of stronger genotype-by-country interactions should not result in different estimates for *r*_*dm_WC*_ as these are estimated within the same country. We used Limousin breed and weaning weight data, but similar estimates of *r*_*dm_WC*_ for weaning weight are reported in other breeds already included in international evaluations, e.g., Charolais (Pabiou et al., 2014). Reported *r*_*dm*_ values for birth and weaning weight in popular beef cattle breeds (Trus and Wilton, 1988; Meyer et al., 1993; Waldron et al., 1993; Meyer, 1994; Dodenhoff et al., 1999; Phocas and Laloë, 2004), on average, are close to the *r*_*dm_WC*_ values observed for weaning weight in Limousin, albeit there are large differences between estimates across studies. Nevertheless, assuming that the absolute *r*_*dm_BC*_ for such breeds is smaller than *r*_*dm_WC*_ as it was in this study suggests that in international evaluations of weight traits for several other beef breeds it may also be valid to assume that *r*_*dm_BC*_ are zero. For a trait like yearling weight, however, reported *r*_*dm*_ values are typically stronger (over ±0.55) (Waldron et al., 1993; Meyer, 1994; Robinson, 1996b), suggesting that both *r*_*dm_WC*_ and *r*_*dm_BC*_ may be stronger than for weaning weight, possibly to the extent that in those cases it would be needed to use estimated *r*_*dm_BC*_ in international evaluations. *r*_*dm_WC*_ estimated at a national level may be biased due to data structure or modeling issues as reported in the literature (Meyer, 1997; Clément et al., 2001; Bijma, 2006). This could potentially also affect Interbeef evaluations as the current Interbeef AMACI model adopts each participating countries’ national model. The results of this study show that *r*_*dm_WC*_ have an impact on the re-ranking of IEBVs, underlining the importance of validation procedures of participating countries’ national models.

Domestic animals’ EBV are expected to re-rank when data from relatives recorded in other countries are accounted for through international evaluations (Venot et al., 2014). In popular breeds such as Limousin, including information from countries as France where most connections and founders can be traced (Bouquet et al., 2011; Bonifazi et al., 2020b) is beneficial for two reasons as shown in previous studies (Venot et al., 2014; Bonifazi et al., 2020a). First, accounting for information from relatives recorded in other countries leads to higher EBV’s reliabilities of domestic animals, especially for smaller countries. Second, breeders get access to elite foreign bulls IEBV expressed on their own country scale and that rank similar or higher than domestic ones, giving breeders the opportunity to achieve higher genetic gains and better meet their selection objectives (Bonifazi et al., 2020a).

The LR method has been applied in evaluations with multiple traits, maternally affected traits, and traits where phenotypes are not available on all individuals for all environments (Chu et al., 2019; Macedo et al., 2020; Picard Druet et al., 2020). As such, the LR method appears to be a useful choice to evaluate the increase in population accuracy when ignoring *r*_*dm*_ in the context of beef cattle international evaluations. The LR statistics $\rho_{p,w}$ relies on two assumptions (Legarra and Reverter, 2018). The first assumption, which does not affect $\rho_{p,w}$, is that the regression of ${\hat{u}}_{p}$ and of ${\hat{u}}_{w}$ on the TBV is equal to one. The second assumption is that the regression of ${\hat{u}}_{w}$ on ${\hat{u}}_{p}$ is also equal to one, i.e., $cov\left({\hat{u}}_{w},{\hat{u}}_{p}\right)=var\left({\hat{u}}_{p}\right)$. When the LR method was applied, this latter assumption did hold for most of the comparisons between scenarios as shown by the regression coefficients ${\hat{b}}_{w,p}$ being mostly between 0.9 and 1.1. We further noticed that the assumption of the LR method was met very closely, especially for domestic animals with reliable EBVs under the evaluation *p* (i.e., with country recorded phenotypes and with direct or maternal REL for evaluation *p* > 0.6; results not shown). Recently, Macedo et al. (2020) reported that some of the LR method statistics may be sensitive to incorrectly estimated genetic parameters; nonetheless, the same authors reported that the ratio of population accuracies $\rho_{p,w}$ performed well in their study. Results of the reported ${\hat{\rho }}_{p,w}$ statistic should be treated with caution since estimates of *r*_*g*_ between countries are prone to sampling error during the estimation process and often reported with high SE (Bonifazi et al., 2020b). Nevertheless, we expect that the observed trends in increases of population accuracy across the scenarios are not affected by departures of the underlying assumptions of the LR method or inaccuracy of estimated variance components.

## Conclusions

Results of this study, based on Limousin weaning weight data, provide support that the current practice of ignoring *r*_*dm_BC*_ in international beef cattle evaluations results in limited decreases in population accuracies, negligible impact on dispersion of EBVs, and no or limited re-ranking of animals’ direct and maternal IEBVs, respectively. Re-ranking for maternal IEBVs was mainly related to animals with REL ≤ 0.3. No re-ranking was present for sires with publishable IEBVs. Moreover, results show that fixing *r*_*dm_WC*_ to 0 would result in considerable re-ranking of animals.

## Supplementary Material

skab222_suppl_Supplementary_Materials

## Abbreviations

CB: common bulls
EBV: estimated breeding value
IEBV: international estimated breeding value
LR: linear regression
REL: approximated reliability
rg: genetic correlations
rdm: direct-maternal genetic correlations
rdm_WC: within-country direct-maternal genetic correlations
rdm_BC: between-country direct-maternal genetic correlations
SD: standard deviation
