# Supplementary material for: The impact of direct-maternal genetic correlations on international beef cattle evaluations for Limousin weaning weight
Source: J Anim Sci. 2021 Aug 1;99(9):skab222. doi: 10.1093/jas/skab222 (PMC8442942; doi:10.1093/jas/skab222)
Supplement: skab222_suppl_Supplementary_Materials [file skab222_suppl_supplementary_materials.docx]

# Supplementary file 1: Table S1-S5

Table S1. List of fixed, random and covariate environmental effects in the national model per each country.

Table S2. National genetic, environmental and residual (co)variances.

Table S3. Number of animals in each class of reliability (REL)­ per country for direct (Dir) and maternal (Mat) international estimated breeding values (IEBV).

Table S4. Distribution of publishable sires’ individual reliabilities (REL) per country.

Table S5. Top 100 sires absolute mean rank position change between scenarios for direct and maternal international estimated breeding values (IEBV).

Table S1. List of fixed, random and covariate environmental effects in the national model per each country ^2^.

| COU ^1^ | Fixed | | | | | | | Random | | | Covariates | |
| --- | --- | --- | --- | --- | --- | --- | --- | --- | --- | --- | --- | --- |
| CZE |  | asextwin | aaca | year |  |  |  | PE | HYS |  |  |  |
| DFS | HYS | asex | aaca | seas | twin |  |  | PE |  |  |  |  |
| ESP | herd_birth | asex | aaca |  |  |  |  | PE |  |  |  |  |
| GBR | HYS_mgt | asex |  | month | twin | fostered | wdam_brd | PE |  |  | agedam | agedam2 |
| IRL | HYS | asex | pariagedam |  |  |  |  | PE |  |  | agedam2 | aawg |
| FRA | HY-asex-mgt |  | pariaaca | seas |  |  | individual | PE |  |  |  |  |
| DEU |  | asex | parity | month | twin |  |  |  | HY |  |  |  |
| CHE |  | asex |  | yearmonth |  |  | alpine | PE | HY | SireHerd | agedam | agedam2 |

^1^ Country = CZE = Czech Republic, DFS = Denmark, Finland and Sweden, ESP = Spain, GBR = Great Britain, IRL = Ireland, FRA = France, DEU = Germany, CHE = Switzerland. ^2^ *aaca* = age at calving; *aawg* = age at weighting; *agedam* = age of the dam; *agedam*2 = age of the dam fitted as quadratic effect; *alpine* = access to alpine grazing for calves; *asex* = sex of the animal; *asextwin* = interaction between *asex* and *twin*; *fostered* = foster code; *herd_birth* = contemporary group defined based on the herd and birth date; *HY* = Herd-Year; *HY-asex-mgt* = contemporary group defined based on *HY*, *asex* and management group defined as calf‐dam couple; *HYS* = Herd-Year-Season; *HYS_mgt* = contemporary group defined by herd, management group and date of birth; *individual* = individual situation, e.g. preferential treatment; *month* = month of birth; *pari* = parity; *pariaaca* = interaction between *pari* and *aaca* effects; *pariagedam* = interaction between *pari* and *agedam*; *PE* = maternal permanent environmental effect; *seas* = season; *SireHerd* = interaction between sire and herd; *twin* = twinning; *wdam_brd* = breed of the weaning dam; *year* = year of birth; *yearmonth* = interaction between *year* and *month*. List originally reported in Bonifazi et al. [37].

Table S2. National genetic, environmental and residual (co)variances ^2^.

| **COU ^1^** | **σ^2^_HYS_** | **σ^2^_HY_** | **σ^2^_Sire-Herd_** | **σ^2^_PE_** | **σ^2^_dir_** | **σ^2^_mat_** | **σ_dir-mat_** | **σ^2^_res_** | **h^2^_dir_** | **h^2^_mat_** |
| --- | --- | --- | --- | --- | --- | --- | --- | --- | --- | --- |
| CZE | 1782 |  |  | 81 | 310 | 197 | -28.53 | 374 | 0.11 | 0.07 |
| DFS |  |  |  | 90 | 269 | 120 | -24.69 | 547 | 0.27 | 0.12 |
| ESP |  |  |  | 43 | 136 | 68 | -21.18 | 294 | 0.26 | 0.13 |
| GBR |  |  |  | 63 | 268 | 55 | -11.63 | 421 | 0.34 | 0.07 |
| IRL |  |  |  | 45 | 450 | 194 | -55.13 | 647 | 0.35 | 0.15 |
| FRA |  |  |  | 63 | 242 | 62 | -40.56 | 354 | 0.36 | 0.09 |
| DEU |  | 477 |  |  | 383 | 326 | -86.43 | 719 | 0.21 | 0.18 |
| CHE |  | 142 | 86 | 69 | 130 | 54 | 33.52 | 565 | 0.12 | 0.05 |

^1^ Country: CZE = Czech Republic, DFS = Denmark, Finland and Sweden, ESP = Spain, GBR = Great Britain, IRL = Ireland, FRA = France, DEU = Germany, CHE = Switzerland. ^2^ σ^2^ = variance, HYS = Herd-Year-Season, HY = Herd-Year, Sire-Herd = interaction between Sire and Herd, PE = maternal permanent environment, h^2^ = heritability, dir = direct genetic effect, mat = maternal genetic effect, σ_dir-mat_ = direct-maternal genetic covariance, res = residual.

Table S3. Number of animals in each class of reliability (REL)­ ^2^ per country for direct (Dir) and maternal (Mat) international estimated breeding values (IEBV).

|  | **Number of animals** | | | | | |
| --- | --- | --- | --- | --- | --- | --- |
| **COU ^2^** | **REL ≤ 0.3** | | **0.3 < REL ≤ 0.6** | | **0.6 < REL** | |
|  | Dir | Mat | Dir | Mat | Dir | Mat |
| CZE | 720,516 | 2,943,083 | 2,702,478 | 485,017 | 8,748 | 3,642 |
| DFS | 228,329 | 3,277,750 | 3,144,370 | 152,181 | 59,043 | 1,811 |
| ESP | 598,794 | 3,247,000 | 2,820,177 | 183,977 | 12,771 | 765 |
| GBR | 313,252 | 2,857,086 | 3,084,474 | 570,358 | 34,016 | 4,298 |
| IRL | 724,382 | 3,001,151 | 2,699,170 | 428,715 | 8,190 | 1,876 |
| FRA | 195,449 | 2,121,322 | 2,932,768 | 1,284,177 | 303,525 | 26,243 |
| DEU | 363,291 | 3,230,289 | 3,038,454 | 185,189 | 29,997 | 16,264 |
| CHE | 2,184,863 | 3,164,338 | 1,244,741 | 266,977 | 2,138 | 427 |

^1^ REL computed under Scenario REF: both *r_dm_WC_* (within-country direct-maternal genetic correlations) and *r_dm_BC_* (between-country direct-maternal genetic correlations) used in the evaluation. ^2^ Country: CZE = Czech Republic, DFS = Denmark, Finland and Sweden, ESP = Spain, GBR = Great Britain, IRL = Ireland, FRA = France, DEU = Germany, CHE = Switzerland.

Table S4. Distribution of publishable sires’ individual reliabilities (REL) ^1^ per country.

| Effect | COU ^2^ | Min. | 1st Qu. | Median | Mean | 3rd Qu. | Max. |
| --- | --- | --- | --- | --- | --- | --- | --- |
| Direct | CZE | 0.38 | 0.53 | 0.56 | 0.57 | 0.60 | 0.97 |
|  | DFS | 0.43 | 0.70 | 0.74 | 0.74 | 0.78 | 0.99 |
|  | ESP | 0.38 | 0.54 | 0.57 | 0.59 | 0.61 | 0.98 |
|  | GBR | 0.34 | 0.60 | 0.64 | 0.65 | 0.68 | 0.99 |
|  | IRL | 0.30 | 0.52 | 0.56 | 0.56 | 0.59 | 0.99 |
|  | FRA | 0.33 | 0.82 | 0.87 | 0.85 | 0.91 | 1.00 |
|  | DEU | 0.33 | 0.59 | 0.63 | 0.64 | 0.67 | 0.98 |
|  | CHE | 0.35 | 0.47 | 0.51 | 0.51 | 0.54 | 0.97 |
| Maternal | CZE | 0.25 | 0.48 | 0.53 | 0.53 | 0.58 | 0.97 |
|  | DFS | 0.20 | 0.34 | 0.38 | 0.40 | 0.43 | 0.97 |
|  | ESP | 0.21 | 0.37 | 0.41 | 0.42 | 0.46 | 0.96 |
|  | GBR | 0.20 | 0.50 | 0.56 | 0.56 | 0.62 | 0.98 |
|  | IRL | 0.20 | 0.45 | 0.50 | 0.50 | 0.55 | 0.97 |
|  | FRA | 0.26 | 0.64 | 0.70 | 0.70 | 0.77 | 1.00 |
|  | DEU | 0.20 | 0.35 | 0.39 | 0.41 | 0.44 | 0.97 |
|  | CHE | 0.21 | 0.41 | 0.45 | 0.45 | 0.50 | 0.95 |

^1^ REL computed under Scenario REF: both *r_dm_WC_* (within-country direct-maternal genetic correlations) and *r_dm_BC_* (between-country direct-maternal genetic correlations) used in the evaluation. ^2^ Country: CZE = Czech Republic, DFS = Denmark, Finland and Sweden, ESP = Spain, GBR = Great Britain, IRL = Ireland, FRA = France, DEU = Germany, CHE = Switzerland.

Table S5. Top 100 sires ^1^ absolute mean rank position change between scenarios ^2^ for direct and maternal international estimated breeding values (IEBV).

|  | |  | **Absolute mean rank**  **position change** | |
| --- | --- | --- | --- | --- |
| **Scenario ^2^** | | **COU ^3^** | **Direct** | **Maternal** |
| REF | CUR | CZE | 1.7 | 5.8 |
|  |  | DFS | 2.2 | 10.1 |
|  |  | ESP | 1.4 | 2.1 |
|  |  | GBR | 1.5 | 4.8 |
|  |  | IRL | 1.0 | 3.4 |
|  |  | FRA | 0.0 | 1.4 |
|  |  | DEU | 2.7 | 8.3 |
|  |  | CHE | 4.7 | 3.0 |
| REF | NONE | CZE | 16.1 | 30.7 |
|  |  | DFS | 13.0 | 13.1 |
|  |  | ESP | 14.3 | 37.7 |
|  |  | GBR | 9.5 | 8.6 |
|  |  | IRL | 11.7 | 13.0 |
|  |  | FRA | 0.4 | 23.2 |
|  |  | DEU | 22.1 | 22.8 |
|  |  | CHE | 42.2 | 34.7 |

^1^ Top 100 publishable sires obtained under Scenario REF. ^2^ Scenario: NONE = both *r_dm_WC_* and *r_dm_BC_* set to 0, CUR = *r_dm_WC_* used in the evaluation, and *r_dm_BC_* set to 0, REF = both *r_dm_WC_* and *r_dm_BC_* used in the evaluation. With *r_dm_WC_* = within-country direct-maternal genetic correlations, and *r_dm_BC_* = between-country direct-maternal genetic correlations. ^3^ COU = Country: CZE = Czech Republic, DFS = Denmark, Finland and Sweden, ESP = Spain, GBR = Great Britain, IRL = Ireland, FRA = France, DEU = Germany, CHE = Switzerland.
